# Is Trait Rumination Associated with the Ability to Generate Effective Problem Solving Strategies? Utilizing Two Versions of the Means-Ends Problem-Solving Test

**DOI:** 10.1007/s10942-015-0227-6

**Published:** 2015-11-19

**Authors:** Akira Hasegawa, Haruki Nishimura, Yuko Mastuda, Yoshihiko Kunisato, Hiroshi Morimoto, Masaki Adachi

**Affiliations:** Faculty of Human Relations, Tokai Gakuin University, 5-68 Naka-kirino, Kakamigahara City, Gifu 504-8511 Japan; Graduate School of Comprehensive Human Sciences, University of Tsukuba, 1-1-1 Tennodai, Tsukuba, Ibaraki 305-8577 Japan; Faculty of Education, Hirosaki University, 1 Bunkyo, Hirosaki City, Aomori 036-8560 Japan; Department of Psychology, School of Human Sciences, Senshu University, 2-1-1, Higashimita, Tama-ku, Kawasaki-shi, Kanagawa 214-8580 Japan; Faculty of Psychology, Hiroshima International University, 555-36 Kurose-gakuendai, Higashi-hiroshima, Hiroshima 739-2695 Japan; Graduate School of Medicine, Hirosaki University, 5 Zaifu-cho, Hirosaki City, Aomori 036-8562 Japan

**Keywords:** Depressive rumination, Depression, Social problem solving, Ruminative Responses Scale, Means-Ends Problem-Solving Test

## Abstract

This study examined the relationship between trait rumination and the effectiveness of problem solving strategies as assessed by the Means-Ends Problem-Solving Test (MEPS) in a nonclinical population. The present study extended previous studies in terms of using two instructions in the MEPS: the second-person, actual strategy instructions, which has been utilized in previous studies on rumination, and the third-person, ideal-strategy instructions, which is considered more suitable for assessing the effectiveness of problem solving strategies. We also replicated the association between rumination and each dimension of the Social Problem-Solving Inventory-Revised Short Version (SPSI-R:S). Japanese undergraduate students (*N* = 223) completed the Beck Depression Inventory-Second Edition, Ruminative Responses Scale (RRS), MEPS, and SPSI-R:S. One half of the sample completed the MEPS with the second-person, actual strategy instructions. The other participants completed the MEPS with the third-person, ideal-strategy instructions. The results showed that neither total RRS score, nor its subscale scores were significantly correlated with MEPS scores under either of the two instructions. These findings taken together with previous findings indicate that in nonclinical populations, trait rumination is not related to the effectiveness of problem solving strategies, but that state rumination while responding to the MEPS deteriorates the quality of strategies. The correlations between RRS and SPSI-R:S scores indicated that trait rumination in general, and its brooding subcomponent in particular are parts of cognitive and behavioral responses that attempt to avoid negative environmental and negative private events. Results also showed that reflection is a part of active problem solving.

## Introduction

Depressive rumination is defined as “behaviors and thoughts that focus one’s attention on one’s depressive symptoms and on the implications of these symptoms” (Nolen-Hoeksema 1991, p. 569). Many experimental and questionnaire-based studies have shown that rumination increases depression. For example, negative mood increased in dysphoric and depressed participants that were asked to ruminate about their current feelings for 8 min, whereas participants that were induced to distract from thoughts and feelings improved their mood (Nolen-Hoeksema and Morrow 1993; Lyubomirsky and Nolen-Hoeksema 1995; Lyubomirsky et al. 1999; Donaldson and Lam 2004; Lavender and Watkins 2004). Moreover, higher total scores on the Ruminative Responses Scale (RRS; Nolen-Hoeksema and Morrow 1991) were predictive of more severe depression, and with the onset and relapse of major depressive episodes (Nolen-Hoeksema and Morrow 1991; Nolen-Hoeksema 2000; Spasojevic and Alloy 2001).

Subsequently, Treynor et al. (2003) extracted two factors from the RRS: *brooding*, which is “a passive comparison of one’s current situation with some unachieved standard,” and *reflective pondering* or *reflection*, which is “a purposeful turning inward to engage in cognitive problem solving to alleviate one’s depressive symptoms” (Treynor et al. 2003, p. 256). Many cross-sectional studies showed that brooding was correlated more strongly with depression than reflection (Treynor et al. 2003; Schoofs et al. 2010; Hasegawa 2013). In addition, several longitudinal studies demonstrated that brooding was associated with more depression at 6 months to 1 year later, whereas reflection was associated with less depression or was not associated (Treynor et al. 2003; Pearson et al. 2010; Schoofs et al. 2010; except for Hasegawa et al. 2015a). These findings suggest that brooding may represent a maladaptive aspect of rumination, whereas reflection may be adaptive or less maladaptive.

One possible process by which rumination, and especially its brooding subcomponent intensifies depression, whereas the reflection subcomponent reduces, or remains unassociated with depression might be through the effects of rumination and reflection on social problem solving (Nolen-Hoeksema 1991; Treynor et al. 2003). Social problem solving is the process through which people attempt to identify or discover effective and adaptive solutions to problems they experience in everyday life (D’Zurilla et al. 2002).

Studies have often utilized two measures of social problem solving: Means-Ends Problem-Solving Test (MEPS; Platt and Spivack 1975) and Social Problem-Solving Inventory-Revised (SPSI-R; D’Zurilla et al. 2002), or a short version of the SPSI-R (SPSI-R:S; D’Zurilla et al. 2002). The MEPS is a performance measure that assesses the effectiveness of problem solving strategies. The SPSI-R or SPSI-R:S are self-report measures assessing five dimensions of the problem solving processes: positive problem orientation (PPO), negative problem orientation (NPO), rational problem solving (RPS), impulsivity/carelessness style (ICS), and avoidance style (AS).

Previous studies showed that in nonclinical samples, the correlations between scores on RRS total scale and subscales and the MEPS scores were not statistically significant (Donaldson and Lam 2004) or very small (.08 < *r* < .17; Hasegawa et al. 2015b). In addition, RRS total scale or brooding subscale scores were positively associated with NPO and AS (McMurrich and Johnson 2008; Hasegawa et al. 2015b). On the other hand, reflection was positively associated with PPO, and more strongly correlated with RPS as compared to brooding (Hasegawa et al. 2015b).

Hasegawa et al. (2015b) also demonstrated that the NPO, ICS, and AS were positively correlated with depression and the PPO was negatively correlated with depression. These findings suggest that one pathway through which rumination leads to depression in nonclinical populations could be through increasing the negative problem orientation, and the avoidance problem solving style. Rumination may lead to negative interpretations about the self, about one’s situation, and future events (Lyubomirsky and Nolen-Hoeksema 1995; Lyubomirsky et al. 1999; Lavender and Watkins 2004). These negative interpretations could activate avoidance goals (such interpretations and avoidance goals correspond to negative problem orientation) and avoidance goals could lead to an avoidance problem solving style that prevents a person from solving his or her problems, which would lead to the deterioration of his or her environment, as well as to sustained rumination about problems or about the self. Providing support to this argument, it has been demonstrated that the total RRS score was positively associated with avoidance behaviors (Kanter et al. 2007; Moulds et al. 2007), and with concerns over mistakes in perfectionism (Harris et al. 2008). The association of reflection with PPO and RPS also suggests that reflection is a part of active problem solving.

The findings described above provide useful information about the process of depressive ruminations. However, further investigation about the relations between rumination and social problem solving are required. The present study replicated and extended the study that examined the association between rumination and social problem solving. In this study, we first investigated the association between measures of rumination and two versions of MEPS scores, and next reexamined the association between measures of rumination and SPSI-R:S.

Although previous cross-sectional studies showed the nonsignificant or weak associations between scores on the RRS and MEPS in nonclinical populations (Donaldson and Lam 2004; Hasegawa et al. 2015b), other studies have reported negative associations between RRS scores and MEPS. For example, it has been shown that the total RRS score was negatively correlated with MEPS effectiveness in two depressed populations (*r* ranged from −.49 to −.53; Donaldson and Lam 2004; Raes et al. 2005). In addition, each MEPS score decreased in dysphoric and depressed participants that were induced to ruminate compared to participants that were induced to distract (Lyubomirsky and Nolen-Hoeksema 1995; Lyubomirsky et al. 1999; Donaldson and Lam 2004).

These findings indicate that in nonclinical populations, *trait rumination* is not related to the quality of responses to the MEPS, whereas *state rumination* while responding to the MEPS deteriorates the quality of solutions. Here, trait rumination refers to ruminative tendencies, or the frequency of rumination experienced in daily life that are assessed by the RRS, whereas state rumination refers to momentary rumination that is manipulated by the rumination induction method developed by Nolen-Hoeksema and Morrow (1993). Findings from nonclinical and clinical populations have also indicated that clinically depressed participants with high ruminative tendencies easily reactivate ruminative thoughts; and therefore they ruminate while responding to scenarios in the MEPS, which are trivial dysphoric experiences. Ruminating while responding to the MEPS might deteriorate the quality of solutions, resulting in the significant negative correlations between total RRS score and MEPS effectiveness observed in clinical samples (Donaldson and Lam 2004; Raes et al. 2005).

However, further evidence is required before drawing firm conclusions about the associations between rumination scores and MEPS scores. Because there are different versions of the MEPS that have different instructions, it is necessary to use different instructions and compare results. Some studies including those described above (Lyubomirsky and Nolen-Hoeksema 1995; Lyubomirsky et al. 1999; Hasegawa et al. 2015b) have used the second-person, actual-strategy instructions that require participants to imagine themselves experiencing the situations described in each scenario and respond with the strategies they would implement for identical problems. On the other hand, another study has used the third-person, ideal-strategy instruction requiring participants to describe the ideal strategies that the protagonists of each story would implement to solve the problem situations (e.g., Marx et al. 1992). The latter version was developed to examine the possibility “depressed persons may be able to find ‘ideal’ strategies for another person, as in the third-person instruction, to deal with difficult situations but may not be able to apply these strategies for themselves, as in the second-person instruction” (Marx et al. 1992, p. 78). The instruction to take a third-person perspective and describe ideal strategies would be suitable for assessing the effectiveness of problem solving strategies.

Considering with the suggestion by Marx et al. (1992), the relationship between scores on rumination measures and the MEPS when using third-person, ideal-strategy instruction could demonstrate the pure association between rumination and effectiveness of problem solving strategies. However, previous studies that have examined the association between rumination and social problem solving did not utilize the third-person, ideal-strategy instruction. If the scores on the MEPS when using third-person, ideal-strategy instruction were significantly and negatively associated with rumination measures, whereas scores with second-person, actual-strategy instruction were not, it would be a strong assertion that the ability to generate effective problem solving strategies was related to trait rumination.

Furthermore, the previous studies have not compared the findings obtained with MEPS when using second-person, actual-strategy instruction and third-person, ideal-strategy instruction. Such a comparison would provide valuable information about the type of MEPS instruction that should be utilized in research, and to consider the possibility that different instructions would affect the findings about the MEPS. Therefore, the first purpose of this study was to utilize the two versions of instructions with the MEPS: second-person, actual-strategy instruction, and third-person, ideal-strategy instruction, and compare the associations between MEPS scores obtained by using different instructions with measures of rumination and depression.

The second purpose of the present study was to examine the association between rumination measures and each dimension of the SPSI-R:S. To our knowledge, only two studies have examined the relationship between RRS and SPSI-R or SPSI-R:S scores (McMurrich and Johnson 2008; Hasegawa et al. 2015b). Although the consistent positive correlations between total RRS score and NPO and AS were obtained, the correlation between total RRS score and other SPSI-R subscales were inconsistent. For example, McMurrich and Johnson (2008) showed that total RRS score is positively correlated with ICS (*r* = .30), but Hasegawa et al. (2015b) reported significant but weak correlation (*r* = .14), and the significant correlation disappeared after controlling for depression level. Because the ICS or impulsivity in general are another important factor that predicts depression (Grano et al. 2007; Hasegawa et al. 2015a), it is important to examine whether rumination and ICS are associated with each other or not.

McMurrich and Johnson (2008) excluded currently depressed participants from their study, and about half of their sample had reported a previous history of major depression, it is possible that their data was influenced by the characteristics of their sample. Only one study Hasegawa et al. (2015b) has examined the relationship between scores on the RRS and SPSI-R in a sample of university students with levels of depression similar to that of the general university population. Furthermore, only the study by Hasegawa et al. (2015b) compared the associations of brooding and reflection subscales of the RRS with SPSI-R. Therefore, the present study reexamined these associations in an unselected sample of university students.

The hypotheses of the present study are as follows. First, consistent with previous studies (McMurrich and Johnson 2008; Hasegawa et al. 2015b), it was hypothesized that brooding subscale and total RRS scores would be positively associated with scores on the NPO and AS of the SPSI-R:S, after controlling for depression level. Because findings between total RRS score and scores on other subscales of the SPSI-R (or SPSI-R:S) in previous studies have been inconsistent (McMurrich and Johnson 2008; Hasegawa et al. 2015b), we did not make precise predictions about these correlations. Second, consistent with the report by Hasegawa et al. (2015b), it was hypothesized that the reflection subscale score would be positively correlated with PPO and RPS, and that these relationships would be significant after controlling for depression. We did not make any precise predictions about the associations between rumination measures and MEPS scores, because previous findings have reported controversial findings, as described above. We also compared the results obtained with two versions of the MEPS.

## Method

### Participants

Undergraduate and graduate students from the Hirosaki University, Hiroshima International University, Senshu University, Tokai Gakuin University, and University of Tsukuba in Japan (*N* = 234) took part in the present study between April and July 2014. Students that were under treatment by a psychiatrist, clinical psychologist, or a counselor were excluded from the study for ethical reasons, because of the possibility that their mood could deteriorate as a result of participation in the study. This exclusion criterion limited participants to a “nonclinical population.” Participants received one of two packets of questionnaires distributed randomly: one packet contained the MEPS with instruction to respond with the actual strategy using the second-person perspective (*n* = 119; Actual Strategy Group: ASG), and the other contained the MEPS instructing participants to respond with the ideal strategy using the third-person perspective (*n* = 115; Ideal Strategy Group: ISG).

One participant in the ASG refused to complete the questionnaires after providing informed consent. Data of two participants in the ASG were eliminated due to the missing values. Data of eight participants in the ISG group were eliminated: four participants because of incorrect responses caused by misunderstanding MEPS scenarios, and four because of missing values. Therefore, the final ASG sample composed of 116 participants (38 men, 78 women, mean age 20.03 years, *SD* = 2.06, age range 18–33 years) and the final ISG sample composed of 107 participants (37 men, 70 women, mean age 19.88 years, *SD* = 1.75, age range 18– 27 years). All participants were Japanese except for one Korean woman in the ISG.

### Measures

#### Beck Depression Inventory-Second Edition (BDI-II; Beck et al. 1996)

The BDI-II is a well-validated, 21-item self-report questionnaire that measures the severity of depressive symptoms experienced in the past 2 weeks. Participants rate their responses using a 0–3 scale, with higher scores indicating greater severity of depression. Kojima and Furukawa (2003) translated the BDI-II into Japanese, which has demonstrated good reliability and validity was used in this study. The BDI-II demonstrated high internal consistency (*α* = .89) in the total sample of the present study (*N* = 223).

#### Ruminative Responses Scale (RRS; Nolen-Hoeksema and Morrow 1991)

The RRS includes 22 items, each of which is rated on a 4-point rating scale anchored between 1 (*Almost never*) and 4 (*Almost always*). The RRS consists of five items assessing brooding, and five items assessing reflection, as well as 12 depression-related items. In this scale, brooding and reflection subscale scores and total RRS score are computed. The adequate psychometric properties of the RRS, including good internal consistency and construct validity, and moderate test–retest reliability of the total and subscale scores have been reported (Treynor et al. 2003; Schoofs et al. 2010). The Japanese translation by Hasegawa (2013) was used in this study. Hasegawa (2013) reported that brooding, reflection, and total RRS score have similar internal consistencies and test–retest reliabilities as those of original version. In addition, the correlations between RRS and depression, worry, and self- and external-preoccupation showed that the total scale and subscales of the RRS have adequate convergent and discriminant validity. In the total sample of the present study, the internal consistencies of brooding, reflection and total scale of the RRS were .72, .60, and .88, respectively.

#### Means-Ends Problem-Solving Test (MEPS; Platt and Spivack 1975)

The Japanese version developed by Hasegawa et al. (2015b) was used. In this test, participants are presented with the beginnings and endings of four interpersonal problem situations, which are counterbalanced across participants. Participants are asked to describe in writing, the problem solving strategies that connects the beginnings with the endings of the problem situations. The scenarios utilized by the Japanese version are composed of four situations: (a) you take part in a seminar with unfamiliar classmates and you want to make friends, (b) you realize that a friend is avoiding you, and you try to make up with him or her, (c) you must prepare the material for your presentation in 2 weeks with a shy classmate with whom you have rarely communicated, (d) you argued with the leader of a university circle about club activities, and persuaded him or her to agree with you.

The present study utilized two versions of the MEPS that differed because of the instructions: The second-person, actual-strategy version and third-person, ideal-strategy version. The second-person, actual strategy version has the same instruction as those utilized by Hasegawa et al. (2015b). In this version, participants are asked to imagine themselves experiencing the situations described in each scenario and write down the strategies they would implement if they experienced the same problems. Instruction in the third-person, ideal-strategy version are quite similar to those utilized by Marx et al. (1992). In this version, the participants are presented with stories showing that the protagonist experienced the same problem situations, with endings as those in the actual strategy version. Protagonist of the four stories are named B, C, D, and E, and participants are asked to imagine that all the protagonists are of the same gender as themselves. Then, participants are requested to write down the ideal strategies that the protagonists in each story would implement to solve the problem situations. The present study administered the MEPS by presenting the scenarios and response format on paper.

We assessed “relevant means,” by counting the number of discrete steps that are judged by the rater as being relevant for enabling the protagonist to reach a solution using the criteria outlined by Platt and Spivack (1975) and “effectiveness,” on a rating scale ranging from 1 (*not at all effective*) to 7 (*extremely effective*), as suggested by Marx et al. (1992). The first author, who is an associate professor, and the sixth author who is a full-time lecturer, counted the number of relevant means and scored the effectiveness of the responses. Among the ASG, inter-rater correlations for relevant means ranged from .78 to .89 with a mean of .84, and those for effectiveness ranged from .70 to .80 with a mean of .75. Among the ISG, inter-rater correlations for relevant means ranged from .75 to .88 with a mean of .81, and those for effectiveness ranged from .67 to .77 with a mean of .73. Participants’ scores that represented the mean score between raters were averaged across the four scenarios.

#### Social Problem-Solving Inventory-Revised Short Version (SPSI-R:S; D’Zurilla et al. 2002)

The SPSI-R:S is a 25-item self-report scale designed to measure an individual’s cognitive, affective, and behavioral responses to real life problem solving situations. Each item in the scale is rated on a 5-point rating scale anchored between 0 (*Not at all true of me*) and 4 (*Extremely true of me*). The scale is comprised of five subscales that include positive problem orientation (PPO), negative problem orientation (NPO), rational problem solving (RPS), impulsivity/carelessness style (ICS), and avoidance style (AS). The Japanese translation by Hasegawa et al. (2015b) was utilized in this study. Each subscale of the Japanese SPSI-R:S was correlated with total score on the Problem-Solving Inventory (PSI; Heppner and Petersen 1982), indicating the good concurrent validity (Hasegawa et al. 2015b). Although Hasegawa et al. (2015b) showed that a CFA to examine a five-factor model showed a moderate model fit (CFI = .80, RMSEA = .078), we calculated each subscale score using the same item composition as the original SPSI-R:S, so as to compare findings for the Japanese SPSI-R:S and the original version. The internal consistencies in the total sample of the present study were .78 for PPO, .83 for NPO, .71 for RPS, 74. for ICS, and .79 for AS, respectively.

### Procedure

All participants that agreed to take part in this study completed the packet of questionnaires that included the MEPS, RRS, BDI-II, SPSI-R:S, and another measure that was not related to the purposes of the present study in a group setting. There are no time constraints in answering each questionnaire. When 90 min had passed from the time the participants started making their responses, they were asked if they wanted to continue to respond to the questionnaire then and there, or if they wanted to take the questionnaire back home, complete their responses, and submit it on a later day. The procedure took participants between 30 and 120 min to complete. All participants received a book voucher worth 1000 yen (approximately US $8) for their participation.

### Statistical Analysis

Differences in scores between ASG and ISG for each measure were examined by using a student *t* test and differences in gender composition were examined with a Chi square test. Zero-order Pearson’s correlations were computed between each measure. *Z* tests for independent correlations described by Fisher (1925) were conducted to compare the magnitude of correlations between ASG and ISG. Partial correlations were calculated with BDI-II scores partialled out, in order to examine the direct associations between each score on rumination and social problem solving measures, after controlling for depression.

## Results

There were no significant differences between ASG and ISG for age [*t*(221) = .57, *p* = .57] or sex [*χ*^2^(1, *N* = 223) = .08, *p* = .77]. Descriptive statistics for each scale are displayed in Table 1. The scores on the reflection and total scale of the RRS and RPS were significantly different between ASG and ISG. Group differences in the two types of MEPS scores were non-significant.

**Table 1:** Descriptive statics for each scale in the total sample by group, showing group differences for each scale

	Total sample (*n* = 223)		ASG (*n* = 116)		ISG (*n* = 107)		*t*
	*M*	*SD*	*M*	*SD*	*M*	*SD*	
BDI-II	14.71	9.55	15.37	9.57	13.99	9.53	1.08
Brooding	13.34	3.72	13.76	3.78	12.88	3.61	1.78
Reflection	10.36	3.15	10.89	2.95	9.79	3.27	2.63**
RRS total	51.36	12.20	53.70	12.00	48.83	11.96	3.03**
PPO	8.98	4.45	9.39	4.24	8.53	4.66	1.44
NPO	11.04	4.83	11.52	5.07	10.51	4.53	1.55
RPS	9.58	3.96	10.12	3.65	9.00	4.22	2.13*
ICS	6.02	3.90	5.95	3.87	6.09	3.96	−.28
AS	7.00	4.58	7.03	4.56	6.97	4.62	.10
MEPS relevant means	–	–	3.32	.95	3.20	.96	.92
MEPS effectiveness	–	–	4.25	.53	4.20	.54	.72

Because two versions of the MEPS were utilized, average score of the MEPS relevant means and effectiveness in the total sample were not calculated

*ASG* Actual Strategy Group, *ISG* Ideal Strategy Group, *BDI-II* Beck Depression Inventory-Second Edition, *RRS* Ruminative Responses Scale, *PPO* Positive Problem Orientation, *NPO* Negative Problem Orientation, *RPS* Rational Problem Solving, *ICS* Impulsivity/Carelessness Style, *AS* Avoidance Style, *MEPS* Means-Ends Problem-Solving Test

* *p* < .05; ** *p* < .01

Table 2 showed the correlations between MEPS relevant means and effectiveness, and measures of depression and rumination for each group. Among ASG, only the correlation between MEPS relevant means and BDI-II was significant (*r* = −.19, *p* = .046). Among ISG, neither correlation was significant (*r* ranged from −.13 to .17, *p* > .08). In addition, neither the correlation between MEPS score and measures of depression, nor with rumination differed significantly between ASG and ISG (*Z* values ranged from .01 to −1.86, *p* > .17).

**Table 2:** Correlations between MEPS relevant means and effectiveness, and measures of depression and rumination for each group, with group differences in each correlation

	MEPS relevant means			MEPS effectiveness		
	ASG	ISG	*Z*	ASG	ISG	*Z*
BDI-II	−.19*	−.11	−.57	−.13	−.13	.01
Brooding	−.09	.04	−.89	−.06	.04	−.72
Reflection	−.04	.14	−1.34	−.09	.17	−1.86
RRS total	−.09	.03	−.89	−.08	.03	−.79

*ASG* Actual Strategy Group, *ISG* Ideal Strategy Group, *BDI-II* Beck Depression Inventory-Second Edition, *RRS* Ruminative Responses Scale

* *p* < .05

Partial correlations between both types of MEPS scores and rumination measures were calculated after controlling for BDI-II score. In ASG, partial correlations with MEPS relevant means were −.01 (*p* = .88) for brooding, −.05 (*p* = .60) for reflection, and −.02 (*p* = .85) for RRS total score, and those with MEPS effectiveness were −.01 (*p* = .91) for brooding, −.09 (*p* = .32) for reflection, and −.02 (*p* = .78) for RRS total score. In ISG, partial correlations with MEPS relevant means were .13 (*p* = .20) for brooding, .18 (*p* = .07) for reflection, and .15 (*p* = .13) for RRS total score, and those with MEPS effectiveness were .15 (*p* = .14) for brooding, .21 (*p* = .03) for reflection, and .18 (*p* = .08) for RRS total score.

Table 3 presents the correlations between scores on the self-report measures of depression, rumination, and social problem solving for the total sample. It can be seen that brooding was significantly and positively correlated with reflection (*r* = .36, *p* < .001). Brooding and total RRS scores were significantly and positively correlated with BDI-II (*r* = .49, .56, respectively, *p* < .001), but reflection was not significantly correlated with BDI-II (*r* = .12, *p* = .07). Moreover, BDI-II was positively correlated with NPO and AS (*r* = .52, .32, respectively, *p* < .001), and negatively correlated with PPO (*r* = −.31, *p* < .001). Also, brooding was positively correlated with NPO (*r* = .57, *p* < .001), RPS (*r* = .20, *p* = .003), and AS (*r* = .28, *p* < .001). The correlations between RRS total score and each subscale of the SPSI-R:S had similar values as those for brooding. On the other hand, reflection was positively correlated with PPO and RPS (*r* = .32, 43, respectively, *p* < .001).

**Table 3:** Correlations between self-report measures of depression, rumination, and social problem solving for the total sample (*N* = 223)

	1	2	3	4	5	6	7	8
1. BDI-II	–							
2. Brooding	.49***	–						
3. Reflection	.12	.36***	–					
4. RRS total	.56***	.85***	.60***	–				
5. PPO	−.31***	−.11	.32***	−.03	–			
6. NPO	.52***	.57***	.12	.56***	−.35***	–		
7. RPS	.05	.20**	.43***	.34***	.39***	.18**	–	
8. ICS	.12	.11	.05	.10	.12	.00	−.20**	–
9. AS	.32***	.28***	.09	.33***	−.30***	.51***	.12	.26***

*BDI-II* Beck Depression Inventory-Second Edition, *RRS* Ruminative Responses Scale, *PPO* Positive Problem Orientation, *NPO* Negative Problem Orientation, *RPS* Rational Problem Solving, *ICS* Impulsivity/Carelessness Style, *AS* Avoidance Style, *MEPS* Means-Ends Problem-Solving Test

** *p* < .01; *** *p* < .001

Partial correlations between each RRS score and SPSI-R subscale scores were calculated with BDI-II scores partialled out. It can be seen from Table 4 that brooding was positively associated with NPO (*pr* = .42, *p* < .001), RPS (*pr* = .20, *p* = .003), and AS (*pr* = .15, *p* = .03). Partial correlations between total RRS score and each problem solving scale were quite similar to those for brooding, except for the significant association between total RRS score and PPO (*pr* = .18, *p* = .006). On the other hand, reflection was positively associated with PPO and RPS (*pr* = .38, .43, respectively, *p* < .001).

**Table 4:** Partial correlations between self-report measures of rumination and social problem solving after controlling for BDI-II scores for the total sample (*N* = 223)

	Brooding	Reflection	RRS total
PPO	.05	.38***	.18**
NPO	.42***	.06	.38***
RPS	.20**	.43***	.38***
ICS	.06	.04	.04
AS	.15*	.05	.20**

*RRS* Ruminative Responses Scale, *PPO* Positive Problem Orientation, *NPO* Negative Problem Orientation, *RPS* Rational Problem Solving, *ICS* Impulsivity/Carelessness Style, *AS* Avoidance Style

* *p* < .05; ** *p* < .01; *** *p* < .001

## Discussion

The present study extended the previous studies that examined the association between rumination and social problem solving. In this study, we first investigated the association between measures of rumination and two versions of MEPS scores, and next reexamined the association between measures of rumination and SPSI-R:S.

The present study utilized the two versions of instructions with the MEPS: second-person, actual-strategy instruction, and third-person, ideal-strategy instruction. Using the MEPS with second-person, actual-strategy instruction, neither the correlation nor the partial correlation between rumination and MEPS scores were significant. These findings were similar with those obtained by Hasegawa et al. (2015b) that utilized the same instruction and demonstrated weak correlations between trait rumination and MEPS scores (.08 < *r* < .17), suggesting that the effectiveness of problem solving strategies assessed by the MEPS with second-person, actual-strategy instruction is not related to trait rumination. Along with these results, the scores on the MEPS with third-person, ideal-strategy instruction were not correlated with rumination scores.

Considering with the suggestion by Marx et al. (1992), the third-person, ideal-strategy instruction would be suitable for assessing the effectiveness of problem solving strategies. The relationship between scores on rumination measures and the MEPS when using third-person, ideal-strategy instruction could demonstrate the pure association between rumination and effectiveness of problem solving strategies. The nonsignificant correlations between the scores on the MEPS with third-person, ideal-strategy instruction and RRS scores indicates that the effectiveness of problem solving strategies is unrelated to trait rumination in nonclinical populations. In addition, the present findings, along with previous studies that have induced rumination (Lyubomirsky and Nolen-Hoeksema 1995; Lyubomirsky et al. 1999) indicate that in nonclinical populations, *trait rumination* is not related to the quality of responses to the MEPS, whereas *state rumination* while responding to the MEPS deteriorates the quality of solutions for each scenario.

The present study compared the correlations of measures of depression and rumination with MEPS scores under either of second-person, actual-strategy instruction and the third-person, ideal-strategy instruction. However, magnitudes of the correlations did not differ between two groups with different instructions. In addition, the average score of the MEPS relevant means and effectiveness did not differ significantly between two groups. These findings indicate that the MEPS with the second-person, actual-strategy version and that with third-person, ideal-strategy version assess similar constructs.

However, after controlling for the level of depression, a significant positive correlation was found between reflection and MEPS effectiveness under the third-person, ideal-strategy instruction. This positive association would be consistent with the suggestion by Treynor et al. (2003) that reflection “may eventually be adaptive in reducing negative affect, perhaps because it leads to effective problem solving” (p. 257). Considering the nonsignificant correlation and weak partial correlation, it is necessary to replicate the study on the relationship between reflection and MEPS scores by using third-person, ideal-strategy instruction.

The associations between RRS and SPSI-R:S scores were similar to that obtained by previous studies (McMurrich and Johnson 2008; Hasegawa et al. 2015b). First, total RRS score and brooding subscale score were correlated positively with NPO and AS, and this correlation was significant after controlling for BDI-II score. It is plausible that rumination is a cognitive-behavioral set (i.e., a series of cognitive and behavioral responses) that attempts to avoid negative environmental and negative private events. Moreover, it is also possible that NPO and AS prevents a person from solving his or her problems, and the deterioration of a person’s environment might sustain rumination.

Second, total RRS score and brooding subscale score were positively correlated with RPS. Hasegawa et al. (2015b) indicated the possibility that the RPS, at least in part, reflects ruminative thoughts about problems, because the item content of the RPS includes not only active and constructive problem solving styles, but also the proneness to get information about problems and evaluate past problem situations, which are related to rumination. In addition, many studies have shown nonsignificant correlations between RPS and depression measure (e.g., D’Zurilla et al. 1998; Anderson et al. 2011; see also D’Zurilla et al. 2002).

Third, reflection was positively correlated with PPO and RPS, and this correlation was significant after controlling for BDI-II score. Moreover, using the *Z*-test for the comparison of two overlapping correlations based on dependent groups by Hittner et al. (2003) demonstrated that the association between reflection and RPS was stronger than that between brooding and RPS (*Z* = 3.36, *p* < .001). These findings imply that reflection is a part of active coping with problematic situations, although RPS was not significantly correlated with depression.

The present study showed that neither type of rumination score was correlated with ICS subscale of the SPSI-R:S. This result is generally consistent with that by Hasegawa et al. (2015b). Although McMurrich and Johnson (2008) showed a significant positive correlation between total RRS score and ICS score, their data might have been influenced by the relatively low severity of depressive symptoms, and the high prevalence of prior major depression in their sample. Therefore, we conclude that rumination is not related to ICS in university students. In the 6 months follow-up study by Hasegawa et al. (2015a), of all the subscales of the RRS, SPSI-R:S, and MEPS, only the ICS subscale at Time 1 predicted BDI-II score at Time 2 after controlling for baseline BDI-II score. The above findings by Hasegawa et al. (2015a) as well as present findings indicate that the impulsivity/carelessness style intensified depression via a process that is independent of rumination.

Recently, Carver et al. (2011) showed that impulsive reactivity to emotions could be classified into three aspects. Moreover, Carver et al. (2013) suggested that brooding rumination is a subcomponent of Pervasive Influence of Feelings, one of impulsive reactivity aspects, although they did not assess brooding in their study. Considering with the item content of each scale, ICS assessed in the present study seems to be a subcomponent of the other impulsive reactivity aspect, Feelings that Trigger Action. Therefore, the implication of the study by Carver and his colleagues is consistent with our proposal that rumination and the impulsivity/carelessness style are independent processes.

### Clinical Implications

The present study focused on a nonclinical university student population. However, Tomoda et al. (2000) reported that the one-year prevalence of major depressive disorder based on DSM-IV criteria in a Japanese university population is 20.7 % and that it was 8.9 % in a French university population (Verger et al. 2010). Therefore, many university students seem to suffer from depression. Indeed, about half of the present sample (*n* = 108) scored 14 points or higher on the BDI-II, which is above the cut-off for mild depression (Beck et al. 1996), whereas 8.5 % of the participants (*n* = 19) scored 29 points or higher, which is indicative of a severe level of depression (Beck et al. 1996), even though the participants in this study were limited to a “nonclinical population.” Therefore, there could have been some university students that were in need of treatment and therefore, the findings of this study are considered to have clinical implications for the treatment of dysphoric and ruminative university students.

The present study showed that trait rumination and its brooding subcomponent are related to negative problem orientation and an avoidance problem solving style. The associations between each variable are concurrent relationships, and as a result, causal inferences should be made with caution. However, if rumination, negative problem orientation, and avoidance problem solving style (and avoidance behaviors) interact in nonclinical populations, the intervention that is aimed at decreasing rumination and the avoidance style, and promoting the implementation of the problem solving strategies might be effective for treating depression. Rumination-focused cognitive behaviour therapy (RFCBT; Watkins et al. 2007) is designed to change this cognitive-behavioral vicious cycle. In RFCBT, rumination is conceptualized as a form of abstract, evaluative cognitive processing, which is to be replaced with concrete, process-focused, and specific cognitive processing through treatment. In addition, RFCBT adopts techniques of behavioral activation to decrease avoidance behaviors including rumination, and increase more useful approach behaviors including active problem solving strategies. It is plausible that RFCBT has beneficial effects for dysphoric and ruminative undergraduate students, as well as for patients meeting criteria for medication-refractory residual depression. It is important to investigate causal relationships between rumination, negative problem orientation, and avoidance problem styles, and then to examine the effectiveness of RFCBT for dysphoric and ruminative undergraduate students.

### Limitations

There are some limitations to this study. First, the internal consistency of the reflection subscale was low, although previous studies have demonstrated the good internal consistency of the reflection subscale (e.g., Treynor et al. 2003; Schoofs et al. 2010; Hasegawa 2013). Replication to examine the association between reflection scores and other variables should be required. Second, there were certain significant differences in the scores on measures of rumination and rational problem solving between two groups that received different instructions with the MEPS, which could have influenced the findings of this study. Future investigations should reexamine differences in findings between two groups. Third, the present study was conducted on a sample of university students and did not include a clinical sample. Future studies should include clinical samples and compare associations between rumination and each dimension of social problem solving in clinical samples with nonclinical samples. Forth, the present study examined the concurrent relations between each variable and did not reveal the causal relationships. The longitudinal study or experimental study can show the causal relationships. Finally, the present study divided the participants into two groups and participants in each group completed different versions of the MEPS. The results showed that different versions of the MEPS assessed similar constructs. Moreover, trait rumination and the effectiveness of social problem solving strategies were not significantly related. However, in order to examine whether different instructions influence the construct assessed by MEPS and the association with rumination, it is important that the same participants complete the two different versions of the MEPS. It is suggested that future studies use a within-participants design, although carries the risk that responding to the scale under one set of instruction would bias responses using the other version of instruction.

## Conclusions

This study extended previous studies that have examined the relationship between depressive rumination and social problem solving in nonclinical populations. The present study utilized the two versions of instructions with the MEPS: second-person, actual-strategy instruction which has been utilized in previous studies on rumination, and third-person, ideal-strategy instruction which is considered more suitable for assessing the effectiveness of problem solving strategies. However, trait rumination was not related to deterioration of problem solving strategies assessed by the MEPS, and that this relationship did not change largely as a result of using different instructions with the MEPS. These findings along with the previous findings indicate that in nonclinical populations, *trait rumination* is not related to the quality of responses to the MEPS, whereas *state rumination* while responding to the MEPS deteriorates the quality of solutions. Trait rumination in general, and its brooding subcomponent were positively correlated with a negative problem orientation and an avoidance style, indicating that rumination is a cognitive-behavioral set that attempts to avoid negative environmental and negative private events. Reflection was positively associated with a positive problem orientation and more strongly associated with a rational problem solving style than brooding, indicating that reflection is a part of active problem solving. In spite of certain limitations to this study, these findings provide important information about the processes by which depressive rumination and its brooding subcomponent intensify depression and also suggest how the reflection subcomponent reduces, or remains unassociated with depression. Moreover, the study provides information on the assessment of social problem solving strategies.
